# Evaluation of the cost-effectiveness of bovine brucellosis surveillance in a disease-free country using stochastic scenario tree modelling

**DOI:** 10.1371/journal.pone.0183037

**Published:** 2017-08-31

**Authors:** Viviane Hénaux, Didier Calavas

**Affiliations:** Unité Epidémiologie, Université de Lyon–Agence nationale de sécurité sanitaire de l’alimentation, de l’environnement et du travail (Anses), Lyon, France; Universidad Nacional de la Plata, ARGENTINA

## Abstract

Surveillance systems of exotic infectious diseases aim to ensure transparency about the country-specific animal disease situation (i.e. demonstrate disease freedom) and to identify any introductions. In a context of decreasing resources, evaluation of surveillance efficiency is essential to help stakeholders make relevant decisions about prioritization of measures and funding allocation. This study evaluated the efficiency (sensitivity related to cost) of the French bovine brucellosis surveillance system using stochastic scenario tree models. Cattle herds were categorized into three risk groups based on the annual number of purchases, given that trading is considered as the main route of brucellosis introduction in cattle herds. The sensitivity in detecting the disease and the costs of the current surveillance system, which includes clinical (abortion) surveillance, programmed serological testing and introduction controls, were compared to those of 19 alternative surveillance scenarios. Surveillance costs included veterinary fees and laboratory analyses. The sensitivity over a year of the current surveillance system was predicted to be 91±7% at a design prevalence of 0.01% for a total cost of 14.9±1.8 million €. Several alternative surveillance scenarios, based on clinical surveillance and random or risk-based serological screening in a sample (20%) of the population, were predicted to be at least as sensitive but for a lower cost. Such changes would reduce whole surveillance costs by 20 to 61% annually, and the costs for farmers only would be decreased from about 12.0 million € presently to 5.3–9.0 million € (i.e. 25–56% decrease). Besides, fostering the evolution of the surveillance system in one of these directions would be in agreement with the European regulations and farmers perceptions on brucellosis risk and surveillance.

## Introduction

Surveillance systems of exotic infectious diseases in animals aim to ensure transparency about the country-specific disease situation and to document the officially disease-free status, which guarantees the access to international trade [1]. For highly contagious diseases, surveillance also intends to identify any introductions as early as possible to allow rapid response, and thus limit the public health impact (in the case of zoonotic pathogens) and the financial expenses related to the diffusion of the disease and the implementation of control measures. In absence of outbreak and in a context of decreasing resources for animal health surveillance, the evaluation of the performance and cost of surveillance systems of exotic diseases is required to optimize resource allocation.

France is officially brucellosis-free (OBF) since 2005 in cattle [2]. Bovine brucellosis, due to *Brucella abortus* or *B*. *melitensis*, is a major zoonosis and is responsible for significant reproductive disorders and production losses in cattle. The main clinical sign is abortion, most commonly in the last trimester of gestation. The French bovine brucellosis surveillance and control system was implemented in 1965 when herd prevalence was about 35%. Combining systematic culling of infected herds and vaccination, the system enabled the eradication of the disease and the acquisition of the OBF status. However, the risk of reintroduction is not null as demonstrated by the occurrence of two *B*. *melitensis* outbreaks in 2012 [3]. The first outbreak followed the introduction of an asymptomatic infected bovine from Belgium and the second outbreak originated from a spillover of *B*. *melitensis* from a wild Alpine ibex (*Capra ibex*) population and resulted in two human infections due to raw milk cheese consumption [4, 5]. These two events stress the importance of maintaining a high level of surveillance. The system has not much evolved since its implementation and consists in mandatory notification and investigation of all abortions, annual serological testing of herds and testing of cattle at purchase (introduction controls), with the objective of detecting at least one case in a year if it circulates in the population at a prevalence at most 0.2%. Indeed, the European regulation regarding bovine brucellosis surveillance requires that 99.8% of bovine herds to be recognized as OBF to maintain the country’s status [6]. Several studies evaluated the effectiveness of this surveillance system [7, 8], the performances of abortion surveillance [9, 10], the cost of each surveillance component and the overall cost of the system [11]. These studies underlined a high under-reporting of detected abortions, suggesting a limited sensitivity of clinical surveillance [10]. Besides, the annual cost of the surveillance system was estimated to be 17 million € in 2013 [11] which may be considered high in absence of case. In consequence, there is strong demand for assessing the efficiency of the system (i.e. sensitivity in regards to the costs) to inform decision about potential evolutions.

The goal of this study was to evaluate the cost-effectiveness of the French bovine brucellosis surveillance system. In the context of exotic diseases, sensitivity–defined as the probability that disease (or infection) will be detected if present at a certain level in the host population–is considered as a critical factor of the effectiveness of a surveillance system [12–16]. In a probabilistic framework, it corresponds to 1 minus the probability of a type II error, i.e. the probability that the country is qualified as free of disease while the prevalence is above the threshold level [14]. Timeliness was not considered as a priority indicator as the capacity for early detection of bovine brucellosis is mainly influenced by the limited sensitivity of the bovine abortion surveillance system rather than by the time elapsed between introduction and detection [8]. We used stochastic scenario tree models [13, 15] to compare the efficiency of the current system to alternative surveillance strategies, by relating their performance (system-level sensitivity) to their cost. Particular attention was given to the ratio between expenses paid by cattle owners (for programmed surveillance and introduction controls) and public funds (for abortion surveillance and mitigation measures).

## Materials and methods

The scenario tree method uses a tree structure to describe the population and surveillance components, and to explicitly capture the probability that any animal might be infected with the disease or that it might be detected [17]. The evaluation of the brucellosis surveillance system using scenario tree method included the following steps [18]: 1) Stratification of the cattle population with regard to the risk of brucellosis infection; 2) Identification and description of all surveillance system components (SSC); 3) Quantification of the sensitivity of each SSC; 4) Calculation of the surveillance cost for each SSC; 5) Evaluation of the influence of key parameters; 6) Evaluation of the overall sensitivity (SSe) and cost of alternative combinations of SSC; 7) Identification of the most efficient combination(s).

### Stratification of the population

Trading and movements of live cattle have been widely considered as the main pathway for brucellosis introduction into free cattle herds [19–24]. Given that the movements of quarantined cattle (following a suspicion in a herd) are restricted [6], the threat of brucellosis introduction is posed by the presence of brucellosis in the unrestricted population, i.e. infected herds which have not yet been detected [24]. Therefore, we considered that the risk of disease introduction into a herd was related to the number of bovine purchases (from France or another country). The cattle herd population was divided into three risk groups: *no-trade*, *low-trade* and *high-trade* groups. The proportion of herds in the *no-trade* group corresponded to the annual proportion of holdings which did not introduce any bovine during 2010–2014 (calculated using data from the national cattle register database). The remaining part of the population was divided between the *low-* and *high-trade* groups with equal number of herds. Beef and dairy cattle farms, which represent about 60 and 40% of farms, respectively, were assumed to be equally distributed among risk groups.

The probability of disease introduction into a herd was calculated using the approach developed by [25, 26] and corresponded to the probability that at least one purchased bovine is infected: α = 1 – (1 –*P*) ^*N*^, where *P* is the prevalence of infection in the herd of origin and *N* is the number of introductions into the herd per year. Because all purchased animals come from OBF herds [6], *P* was fixed at 1×10^−5^ [25] for all risk groups. For the *low-* and *high-trade* groups, *N* was set as the first and third quartiles, respectively, of the distribution of the number of animals purchased per herd (excluding herds that did not introduce any bovine). The *no-trade* group was assumed to be the reference (i.e. lowest risk group) and *N* was fixed to 1 (for calculation purposes). For a risk group *g*, the relative risk (RR) was calculated as *RR*_*g*_ = *α*_*g*_/*α*_*NT*_, where *α*_*NT*_ is the probability of disease introduction in the *no-trade* group. Then, the RR were weighted according to the proportion of the population in each group to ensure that the average adjusted risk (AR) for the population was equal to one [15]:

$$\sum_{g=1}^{G}({AR}_{g}\times {Pr}_{g})=1$$ (1)

$$given\;that\;\frac{{AR}_{i}}{{AR}_{j}}=\frac{{RR}_{i}}{{RR}_{j}},$$ (2)

where *G* is the number of groups and *Pr*_*g*_ the proportion of the population belonging to the *g*^th^ group, with *i* and *j* representing specific groups.

### Surveillance system components

SSCs include clinical surveillance (denoted as CLIN hereafter), programmed surveillance (PROG) and introduction (purchase) controls (INTRO). The current CLIN SSC (referred as CLIN1) relies on the mandatory notification and investigation of every abortion. Is considered as an abortion, the expulsion of a fetus or a calf, stillborn or dying less than 48 hours after being born [27]. The probability of an infected bovine having a brucellosis-related abortion was obtained from experimental studies [28–31]; results of those studies were combined following the rules of [32] to obtain mean value and confidence interval. The mean probability of abortion notification by farmers derived from [10] (results from 2006–2011 were combined as described by [32]). It was assumed that all reported abortions were investigated and tested for brucellosis. Abortion investigation consists in blood sampling of the aborted cow to test for *Brucella* spp. using buffered antigen plate agglutination test (BPAT), followed if positive by a complement fixation (CF) test, and then bacteriology (culture). Mean parameter estimates and confidence intervals are given in Table 1.

**Table 1:** Model parameter values used in the stochastic tree scenario analysis to assess the sensitivity (SSe) of the French bovine brucellosis surveillance system.

Parameter	Input value [95% confidence interval]	Sources
Median number of cows (over 24 months-old) per beef herd (excluding herds with ≤ 5 reproductive animals)	36	Data^a^
Median number of cows (over 24 months-old) per dairy herd (excluding herds with ≤ 5 reproductive animals)	52	Data^a^
Within-herd prevalence (P_U_*)	Uniform(1/*n*—0.10)^b^	[4, 33–37]
Proportion of brucellosis-related abortions (%)	63 [30–96]	[28–31]
Proportion of abortions detected (%)	30 [18–42]	[38]
Proportion of abortion notifications in beef cattle (%)	21 [12–29]	[10]
Proportion of abortion notifications in dairy cattle (%)	41 [31–50]	[10]
Probability of infection in purchased bovine	max(P_U_*× P_H_*, 10^−5^)^c^	-
Annual proportion of herds (in the *high-* and *low-trade* groups) with ≥ 1 introduction investigated (%)	47 [29–66]^d^	[11], Data^a^
Annual proportion of introductions investigated per herd (%)	22 [13–30]^d^	[11], Data^a^
BPAT sensitivity (%)	95.4 [89.2–100.0]	[39]
CF test sensitivity (%)	89.0 [64.4–100.0]	[39]
Bacteriology sensitivity (%)	46.1 [28.0–64.2]	[39]
Bulk milk ELISA test sensitivity (%)	97.9 [92.3–100.0]	[39]
Allergic skin-test sensitivity (%)	78.3 [65.6–91.0]	[39]
Milk ring-test sensitivity (%)	89.5 [65.6–100.0]	[39]

^a^ Data from the French national cattle register for 2010–2014

^b^ Uniform distribution ranging from one infected animal per herd (1/*n*) to 0.10

^c^ P_U_*, within-herd prevalence; P_H_*, design prevalence

^d^ Uncertainty interval based on a coefficient of variation of 20%

The current PROG SSC (PROG1) consists in annual testing in all cattle herds [40]. In most dairy herds, testing is conducted on bulk milk sampling. In beef herds (and in dairy herds producing non-pasteurized milk or cheese), testing is conducted on blood samples from 20% of cattle over 24 months-old. Milk samples are tested by milk enzyme-linked immunosorbent assay (ELISA) test, followed, if positive, by a second ELISA on a new milk sample, and then a ring test. Blood serology consists in a BPAT test, followed if positive by a CF test. If positive, a new series of BPAT and CF tests is conducted. Positive serological results lead to allergic skin tests on 20% of bovines of the farm, followed if positive by bacteriology.

Introduction controls (INTRO1) are required for the purchase of animals originating from OBF holdings presenting a particular risk (i.e. holdings with a previous history of brucellosis infection, epidemiologically related to a brucellosis-infected herd or a wildlife reservoir, or not complying with surveillance regulations) or if the transfer between departure and arrival herds exceeds 6 days [40]. These controls are required for animals originating from France or from other countries (whatever the country status regarding bovine brucellosis). We assumed that the probability of infection in introduced bovines was equal to P_H_* × P_U_*. We used data on bovine purchases and purchase controls from the French national cattle register data and the information system of the Ministry of Agriculture for 2013 to calculate the annual proportion of introduced bovines that were tested and the annual proportion of herds (within the *low-* and *high-trade* groups) subject to introduction controls [11]. A coefficient of variation (CV) of 20% was used for calculating confidence intervals. Brucellosis testing consists in blood serology following the same protocol as in PROG1.

Mean diagnostic test sensitivity values (and CV) were extracted from [39]; a CV of 20% was considered for bacteriology, which may be considered as large in comparison to CV values for other diagnostic tests.

### Scenario tree analysis

The sensitivity of detection of a surveillance system is defined as the probability of detecting at least one positive animal for a given prevalence in the herd population, denoted hereafter design prevalence (P_H_*). The choice of P_H_* is not trivial: the lower P_H_*, the higher the level of surveillance needed to demonstrate that the disease is not present [15], but the lower the control measures to eliminate the disease in case of introduction. For bovine brucellosis, the OBF status is maintained if at least 99.8% of herds are demonstrated as free of disease [6]. Accordingly, if brucellosis is detected at a prevalence of 0.2% or higher, economic losses related to movement restrictions and market access limitations (following the loss of the OBF status) will be added to the costs of mitigation. Therefore, the efficiency of the surveillance system was calculated for P_H_* values of 0.1%, 0.05% and 0.02% to evaluate the performances and limits of the current surveillance system, while maintaining a safety margin against the loss of the OBF status.

**Fig 1:**
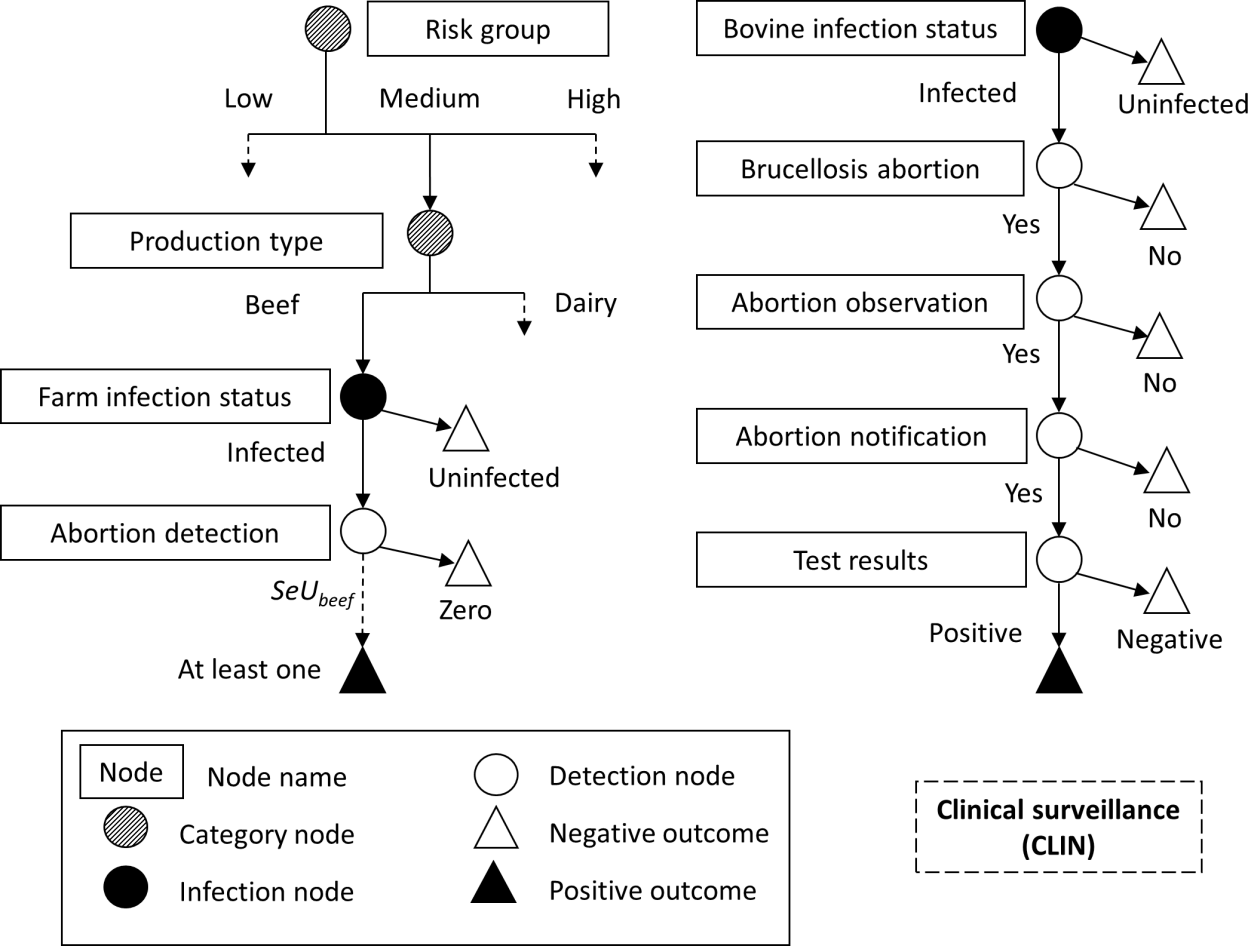
Scenario tree for clinical surveillance (CLIN) for bovine brucellosis in cattle in France, depicting the probability (*CSeU*) that any farm within a risk group and a production type is infected and detected (left), and the probability (*SeU*) that an animal is infected, aborts, and is detected in an infected herd (right). Only the pathway for one of the three risk groups is completed; assume other identical in structure. Screening tests are described in Methods.

**Fig 2:**
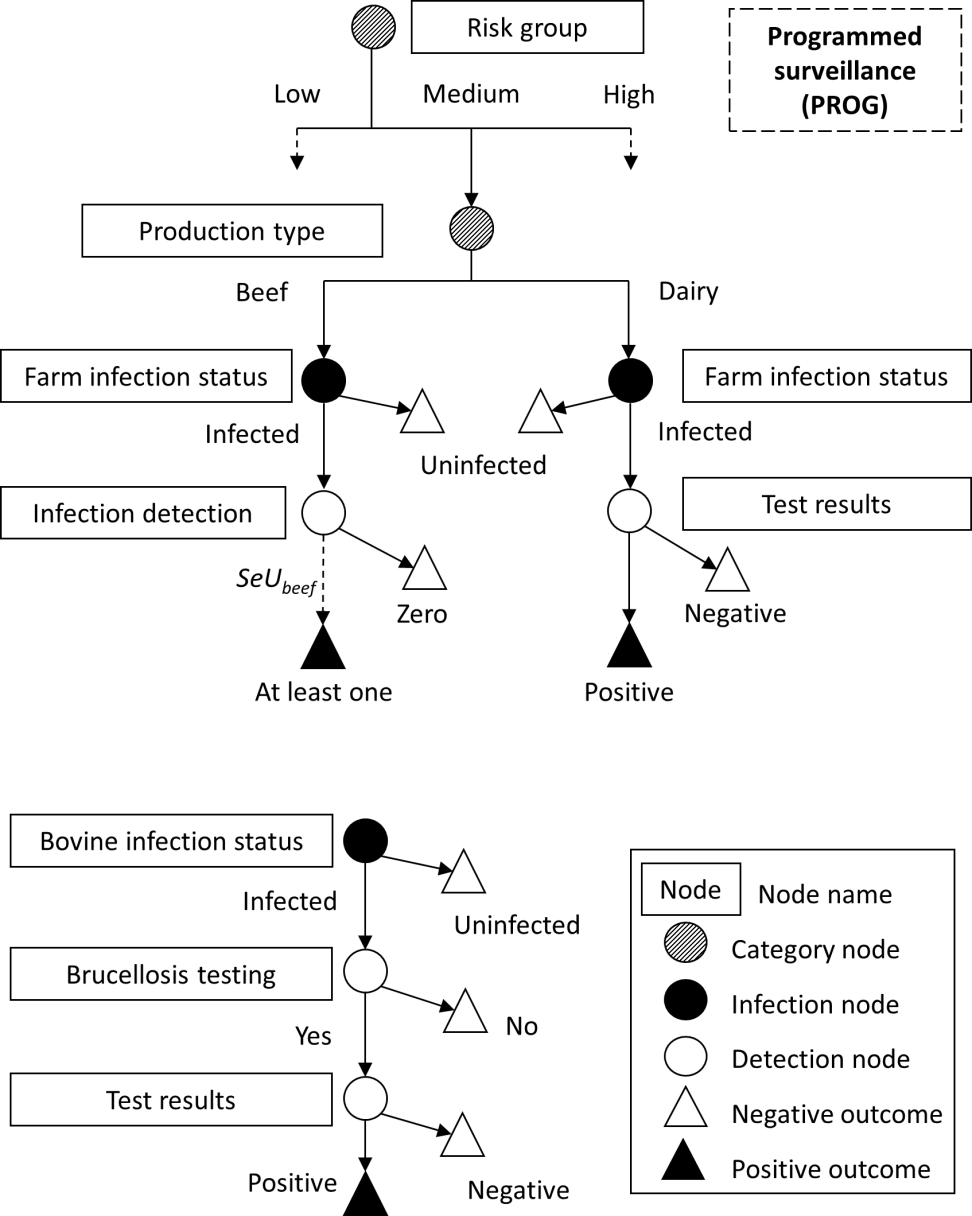
Scenario tree for programmed surveillance (PROG) for bovine brucellosis in cattle in France, depicting the probability (*CSeU*) that any farm within a risk group and a production type is infected and detected (top), and the probability (*SeU*) that an animal is infected, sampled, and detected in an infected beef herd (bottom). Only the pathway for one of the three risk groups is completed; assume other identical in structure. Screening tests are described in Methods.

**Fig 3:**
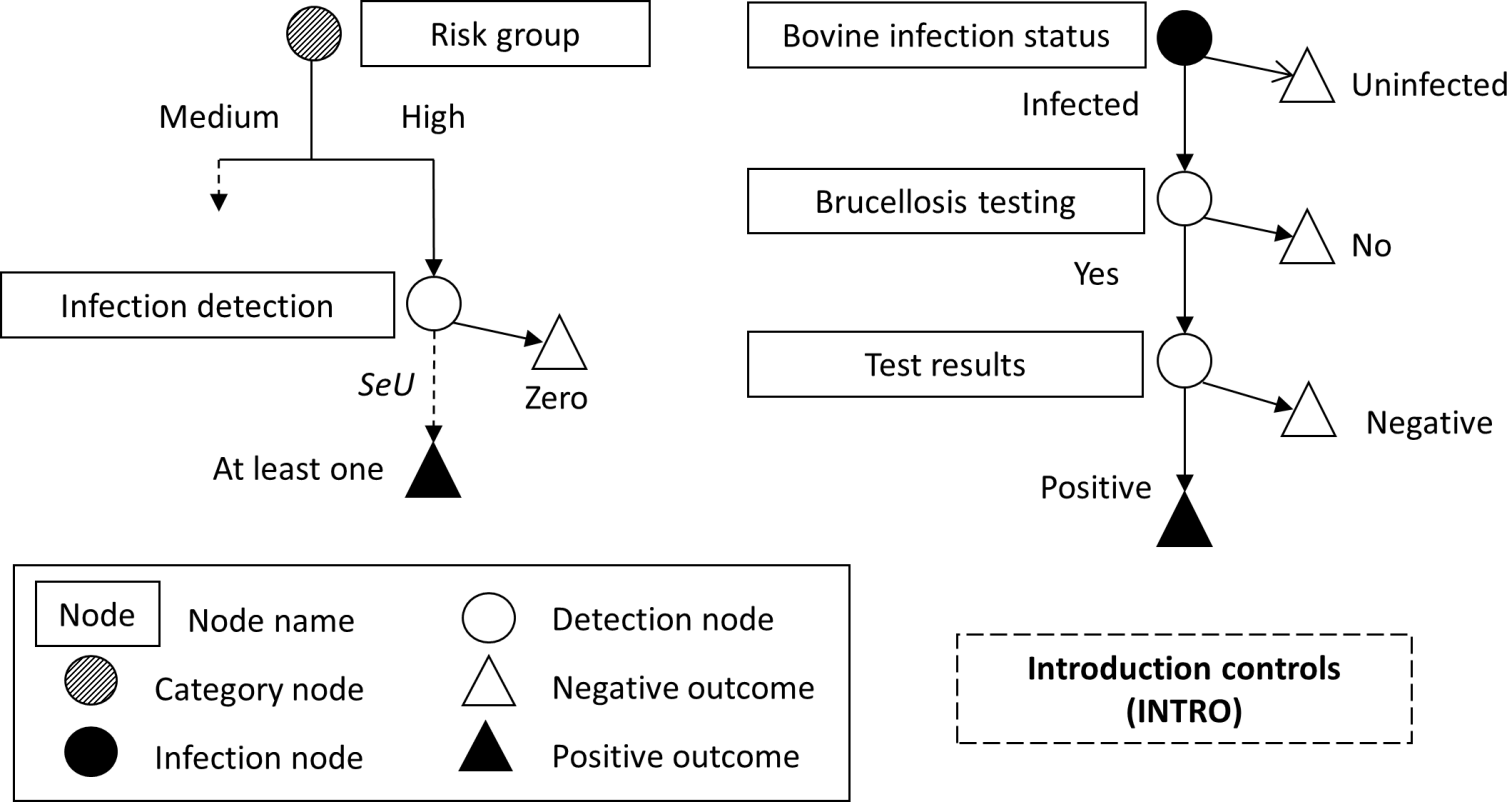
Scenario trees for introduction (purchase) controls (INTRO) for bovine brucellosis in cattle in France, depicting the probability (*CSeU*) that any farm within a risk group is infected and detected (left), and the probability (*SeU*) that an introduced animal is infected, sampled, and detected (right). Only the pathway for one of the three risk groups is completed; assume other identical in structure. Screening tests are described in Methods.

For each SSC, the scenario tree characterizes all possible pathways from the occurrence of an infection to the detection of the case, as a set of events with specified probabilities. Each event is represented by a node dividing the population into groups, within which cattle herds exhibit similar probability of being infected and detected. The sensitivity of each SSC was calculated over a one-year period, based on the scenario trees defined in Figs 1, 2 and 3 and methodology described in [15, 17, 41]. It was assumed that the specificity of the surveillance system was 100%.

For CLIN, the probability that any randomly drawn farm will give a positive outcome (unit sensitivity, *CSeU*) depended on the probability that an infected animal is detected (*SeU*), expressed as the product of the probabilities of brucellosis-induced abortion (*P*_*abort*_), observation of an abortion by the farmer (*P*_*farmer*_), notifying the abortion to the vet (${P_{vet}}_{f}$) (which differs between types of production *f*), and getting positive results at diagnostic tests (*SeU*_*animal*_) given the sampled animal is infected:

$${SeU}_{f}=1-{\left(1-P_{abort}\times P_{farmer}\times {P_{vet}}_{f}\times {SeU}_{animal}\right)}^{n\times P_{U}^{*}},$$ (3)

with $n\times P_{U}^{*}$ the number of infected bovines. We calculated the mean probability of detecting at least one case in any random cattle farm as:

$$SeU=\sum_{f}{Pr}_{f}\times {SeU}_{f}$$ (4)

where *Pr*_*f*_ is the proportion of farms of each production type. For each risk group, the probability that any randomly drawn farm will give a positive outcome was calculated as:

$${CSeU}_{g}=1-{\left(1-SeU\right)}^{N\times {Pr}_{g}\times {AR}_{g}\times P_{H}^{*}}$$ (5)

where *N* × *Pr*_*g*_ was the number of farms falling into the specified risk group g and *AR*_*g*_ × *P*_H_^*^ the associated probability of infection in risk group g. Finally, the sensitivity of the CLIN component of surveillance (*CSe*), corresponding to the probability of detecting at least one brucellosis-positive herd given the presence of brucellosis at a given P_H_*, was calculated as:

$$CSe=1-\prod_{g}{CSeU}_{g}.$$ (6)

For PROG, the sampling and testing procedures vary between types of production. For dairy herds, tested on bulk milk, the probability of detecting brucellosis (*SeU*_*dairy*_) corresponded to the product of test sensitivities. For beef herds, tested by blood serology on a proportion of bovines within the herd, the probability that any randomly tested animal will give a positive result was calculated as:

$${SeU}_{beef}=1-{\left(1-{Pr}_{animal}\times {SeU}_{animal}\right)}^{n\times P_{U}^{*}}$$ (7)

where *Pr*_*animal*_ is the proportion of tested animals, *SeU*_*animal*_ the product of test sensitivities, and $n*P_{U}^{*}$ the number of diseased animals in the herd. Like for CLIN surveillance, the mean probability of detecting bovine brucellosis within a herd was calculated as in (4). Then, for each risk group, the probability that any randomly drawn farm will give a positive outcome was calculated as in (5). At last, the sensitivity of the PROG component of surveillance was obtained using (6).

For INTRO, the probability that any introduced animal will give a positive result was calculated as:

$${SeU}_{g}=1-{\left(1-{SeU}_{animal}\times {Pr}_{animal}\right)}^{n_{g}\times P_{animal}^{*}}$$ (8)

where *SeU*_*animal*_ is the product of test sensitivities, *Pr*_*animal*_ the proportion of introduced (purchased) bovines that are tested per herd and $n_{g}\times P_{animal}^{*}$ the number of introduced brucellosis-infected bovines (with *n*_*g*_ the number of bovine introductions in risk group *g* and $P_{animal}^{*}$ the probability of infection in introduced animals). For the *low-* and *high-trade* groups, the probability that any randomly drawn farm will give a positive outcome was calculated as:

$${CSeU}_{g}={\left(1-{SeU}_{g}\right)}^{N\times {Pr}_{g}\times {Pr}_{z}}$$ (9)

where *Pr*_*z*_ is the proportion of herds (within these two groups) with at least one tested introduction. The sensitivity of INTRO was calculated using (6), with *g* corresponding to the *high-trade* and *low-trade* groups only; the *no-trade* group was not considered as those herds do not introduce bovine.

Assuming that all SSCs are independent, the overall sensitivity of the surveillance system (SSe) was calculated as follows:

$$SSe=1-\prod_{x}\left(1-C{Se}_{x}\right)$$ (10)

where *x* denotes the different SSC.

Model parameters and probabilities for the calculations of the SSC sensitivities are provided in Table 1. Within-herd prevalence ($P_{U}^{*}$) was modelled with a uniform distribution ranging from 1/*n* (i.e. one infected animal per herd) to 0.10 as commonly observed in brucellosis-infected herds [4, 33–37]. Other parameter values were modelled with PERT distributions, using lower and upper values of 95%-confidence (or -credibility) intervals or minimum-maximum values reported in the literature to characterize parameter variability, or a coefficient of variation (CV) of 20% to model uncertainty in absence of data. Mean SSe (and standard error) were estimated using a Monte-Carlo approach with 1,000 simulations in R [42].

### Cost evaluation

For CLIN, the expenses included the cost of the farm visit by the veterinarian following the notification of the abortion, the cost of blood sampling on the aborted cow, and the cost of the detection analyses. The number of abortions per farm depended on the mean number of reproductive females per herd and the probabilities of occurrence, detection, and notification of all-cause abortions (Tables 1 and 2). For PROG on bulk milk, the expenses included only the cost of an ELISA analysis since the collection of milk samples was integrated into the French milk quality program. For PROG on blood and INTRO, the expenses included the costs of the farm visit, commuting time and blood sampling by the veterinarian and the cost of the detection analyses.

**Table 2:** Model parameter values used in the stochastic tree scenario analysis to assess the cost of the French bovine brucellosis surveillance system.

Parameter	Input value (min-max)	Sources
Annual proportion of abortions (all causes included) (%)	7.5 (5–10)	[38, 43]
Vet fee (€) for visit–abortion investigation	27.70	[11]
Vet fee (€) for visit–other cases	24.39 (10.20–41.30)	[11]
Vet fee (€) for commuting time (per km)–abortion investigation	1.24	[11]
Vet fee (€) for commuting time (per km)–other cases	0.60 (0.10–1.20)	[11]
Round-trip distance (km)	15	[11]
Vet fee (€) for blood sampling–abortion investigation	2.77	[11]
Vet fee (€) for blood sampling–other cases	2.30 (1.30–3.10)	[11]
Laboratory cost (€) for BPAT–abortion investigation	3.30 (0.17–4.50)	[11]
Laboratory cost (€) for BPAT–other cases	2.12 (0.17–4.50)	[11]
Laboratory cost (€) for FC test	7.58 (1.70–18.36)	[11]
Laboratory cost (€) for milk ELISA	4.69 (2.82–10.70)	[11]

Given the low P_H_* (under 0.2%) and the high specificity of diagnostic tests (>99%), only few samples were expected to return (true or false) positive results at the detection tests, resulting in a low number of confirmatory tests. For simplification, these costs, considered as negligible, were not included.

Veterinary fees for farm visit, commuting time and blood sampling were extracted from the regulations for animal health (for abortion investigations which are paid by public funds) or from *département*-specific agreements between veterinarians’ and farmers’ representatives (for PROG and INTRO expenses which are paid by farmers) for 2013 (a *département* is a French administrative and territorial division covering a mean surface area of 5,800 km^2^). A round-trip distance of 15km was considered (as mentioned in some agreements). A survey was conducted among *département* veterinary laboratories and inter-professional milk-testing laboratories to obtain the costs of brucellosis-screening analyses for 2013. Veterinary fees and costs of laboratory analyses varied among *départements* and were thus modelled with PERT distributions ranging from the minimum to maximum reported costs (Tables 1 and 2).

### Influence of input parameters on sensitivity and cost of SSC

A sensitivity analysis, using Latin hypercube sampling [44], was conducted to test the potential importance of input parameters on predicted *CSe* and cost of each SSC of the current surveillance system (scenario 1) at P_H_* = 0.02%. This approach assumes that uncertainty in each parameter follows a specific probability distribution. Each parameter distribution is divided into *K* equi-probable segments. A set of parameters for a single run of the model is created by sampling these distributions without replacement to create *K* unique parameter combinations. We set *K* = 100.

For RR, input values were drawn from uniform distributions ranging from the minimum to the median annual number of introductions per herd for the *low-trade* group and from the annual median to a maximum number of 200 introductions for the *high-trade* group. The abortion notification rate may evolve over time depending on farmers’ perceptions of the brucellosis risk and actions implemented to increase farmers’ and veterinarians’ awareness about the need to report abortions. The abortion notification rate was expected to evolve in the same direction in both types of production, so we consider similar notification rate for beef and dairy farmers. The abortion notification rate in dairy farms was modelled using a uniform distribution U[0.01;1]. For other parameters, we used distributions provided in Tables 1 and 2. All parameters were assumed to vary independently.

Linear correlation coefficients (LCC) were used to estimate the correlation between each model parameter value and both *CSe* and cost results. The t-statistic was used to test the significance of the correlation coefficients; p-values were corrected with the Bonferroni approach, as p_Bonferroni_ = N × p_test_, where N is the number of parameters tested for each SSC. The sensitivity analysis was run 100 times to get mean LCC and p-values.

### Alternative SSCs

Several alternatives to the current SSCs were tested. For CLIN, we considered a SSC in which notification would be required after a series of two or more abortions (CLIN2), as bovine brucellosis may cause waves of abortions within infected herds. We calculated the number of expected abortions in an infected herd as: $N_{abortion}=n\times P_{U}^{*}\times P_{abortion}$, where *n* is the number of bovines per herd, $P_{U}^{*}$ the within-herd prevalence and *P*_*abortion*_ the probability that an infected bovine will abort. We assumed that farmers would notify their vet (with the same probability as in CLIN1; Table 1) after two or more abortions and therefore the notification rate was set to 0 when *N*_*abortion*_ was ≤ 1 event. Other parameter values were assumed to be the same as for CLIN1. The cost of CLIN2 was calculated assuming that series of two abortions were notified, considering that about 20% of cattle abortions occur in series of 2 events within 30 days [45]. For PROG, alternatives included: random sampling of 20% of beef herds (tested on blood) with serological testing of all reproductive animals and sampling of all dairy herds (tested on bulk milk) (PROG2), random sampling of 20% of beef herds with serological testing of all reproductive animals and sampling of 20% of dairy herds (PROG3), risk-based sampling (i.e. within the *high-trade* group) of 20% of beef herds with serological testing of all reproductive animals and risk-based sampling of 20% of dairy herds (PROG4), and risk-based sampling of 20% of beef herds with serological testing of 20% of reproductive animals and risk-based sampling of 20% of dairy herds (PROG5). INTRO1 was included or not in scenarios. We used exact or hypergeometric distributions to calculate PROG sensitivity depending on the proportion of herds sampled and the proportion of bovines screened within each herd [17]. In total, 20 scenarios (including the current system) were considered and compared considering their overall sensitivity and cost.

## Results

### Risk groups

During 2010–2014, about 185,000 cattle herds were subject to brucellosis surveillance; fattening herds are exempted of surveillance. Overall, 45% of herds did not introduce any animals and thus composed the *no-trade* group. The remaining herds were divided equally (27.5%) between the *low-* and *high-trade* groups. The RR were estimated to be 1 for the *no-trade* group (reference group), 2 for the *low-trade* group and 15 for the *high-trade* group.

### Sensitivity and cost of each SSC

The estimated sensitivity and cost of each SSC according to P_H_* are provided in Table 3. The mean sensitivity of clinical surveillance was estimated to be 97% for P_H_* = 0.05% if all abortions are notified (CLIN1) and 68% if series of two abortions (CLIN2) were investigated. The sensitivity was predicted to go down to 82% for CLIN1 and 59% for CLIN2 at P_H_* = 0.02%. The cost of CLIN1 was around 2.9 million € and 0.5 million € for CLIN2.

**Table 3:** Mean sensitivity (CSe) and costs (± SE) of alternative surveillance system components (SSC) for bovine brucellosis in France at design prevalence (P_H_*) of 0.10, 0.05% and 0.02%. SSC include clinical surveillance (CLIN), programmed surveillance (PROG), and introduction controls (INTRO)**.**

SSC	CSe—P_H_* = 0.05%^a^	CSe—P_H_* = 0.02%	CSe—P_H_* = 0.01%	Cost (million €)
CLIN1 –notification of every abortion^b^	97.0 ± 4.5	82.1 ± 13.1	60.3 ± 16.1	2.91 ± 0.62
CLIN2 –notification of series of two or more abortions	68.4 ± 44.3	59.1 ± 38.3	44.9 ± 31.7	0.52 ± 0.37
PROG1 –testing of 20% of bovines in 100% of herds^b^^,^^c^	99.5 ± 0.9	91.9 ± 6.6	76.3 ± 10.1	8.61 ± 1.12
PROG2 –beef: testing of 100% of bovines in a random sample of 20% of herds, dairy: sampling of 100% of herds	99.5 ± 0.8	90.9 ± 6.5	73.2 ± 8.9	5.60 ± 0.83
PROG3 –testing of 100% of bovines in a random sample of 20% of herds	97.4 ± 3.8	81.6 ± 11.6	62.0 ± 11.5	5.22 ± 0.81
PROG4 –testing of 100% of bovines in a sample of 20% of herds from the high-risk group	100.0 ± 0.1	98.7 ± 2.5	93.8 ± 6.3	5.28 ± 0.83
PROG5 –testing of 20% of bovines in a sample of 20% of herds from the high-risk group^c^	96.8 ± 3.7	78.4 ± 10.8	57.3 ± 11.1	1.73 ± 0.22
INTRO1 –introduction controls^b^	30.2 ± 13.0	19.2 ± 6.2	17.2 ± 5.0	3.36 ± 0.59

^a^ P_H_*, design prevalence

^b^ SSC of the current surveillance system in France

^c^ The sampling of 20% of cattle within the herd was applied only to blood serology surveillance

The sampling effort (in terms of the overall number of animals tested) was similar in PROG1 and PROG2, resulting in similar sensitivity; yet, the cost of PROG2 was markedly lower (5.6 million €) than the cost of PROG1 (8.6 million €) because of the difference in the proportion of herds tested. The comparison of PROG3 and PROG4 (random vs. risk-based sampling) indicated that for a similar cost (5.2 million €), risk-based sampling was more sensitive. Reducing the proportion of serological tests in high-risk herds from 100 to 20% of reproductive animals (PROG4 vs. PROG5) decreased markedly the sensitivity at P_H_* = 0.02% but not for P_H_* = 0.05% or higher while it cut down the surveillance cost by 67%.

INTRO1 was predicted to have a sensitivity at detecting disease of 44% at P_H_* = 0.1% and 19% at P_H_* = 0.02%. The cost of this SSC was about 3.4 million €.

### Cost-effectiveness

The sensitivity of the current surveillance system (scenario 1) was estimated to be above 98% at P_H_* = 0.02% and above, and 91% at P_H_* = 0.01% for a total cost of 14.9 million € (Table 4). The expenses included 2.9 million € for CLIN1, 3.4 million € for INTRO1 and 8.6 million € for PROG1 (including 8.2 million € for blood serology and 0.4 million € for bulk milk surveillance). Therefore, the cost paid by the private sector (for PROG1 and INTRO1) was about 12.0 million € (79%) versus 2.9 million € covered by public funds (for CLIN1).

**Table 4:** Mean sensitivity and costs (± SE) of alternative surveillance systems for bovine brucellosis in France at P_H_* = 0.02% and P_H_* = 0.05%. At higher P_H_*, all scenarios were predicted to be 100%-sensitive.

Scenarios	SSe—P_H_* = 0.05%^a^	SSe—P_H_* = 0.02%	SSe—P_H_* = 0.01%	Surveillance cost (million €)	Cost (million €) for farmers (%^b^)
1 –CLIN1+PROG1+INTRO1	100.0 ± 0.1	98.6 ± 2.1	91.0 ± 7.0	14.9 ± 1.8	12.0 (81%)
2 –CLIN1+PROG1	100.0 ± 0.1	98.2 ± 2.6	89.3 ± 7.7	11.5 ± 1.3	8.6 (75%)
3 –CLIN1+PROG2+INTRO1	100.0 ± 0.1	98.4 ± 2.1	89.9 ± 6.9	11.8 ± 1.4	9.0 (76%)
4 –CLIN1+PROG2	100.0 ± 0.1	97.9 ± 2.6	88.1 ± 7.8	8.5 ± 1.0	5.6 (67%)
5 –CLIN1+PROG3+INTRO1	99.9 ± 0.3	96.6 ± 4.3	86.1 ± 9.3	11.5 ± 1.4	8.6 (75%)
6 –CLIN1+PROG3	99.9 ± 0.4	95.8 ± 5.0	83.8 ± 10.5	8.2 ± 1.0	5.3 (65%)
7 –CLIN1+PROG4+INTRO1	100.0 ± 0.0	99.8 ± 0.5	97.2 ± 3.8	11.5 ± 1.5	8.6 (75%)
8 –CLIN1+PROG4	100.0 ± 0.0	99.7 ± 0.7	97.0 ± 4.1	8.1 ± 1.1	5.3 (65%)
9 –CLIN1+PROG5+INTRO1	99.9 ± 0.4	96.0 ± 4.6	84.4 ± 9.8	7.2 ± 0.9	4.4 (60%)
10 –CLIN1+PROG5	99.8 ± 0.5	95.4 ± 4.9	81.8 ± 10.8	4.6 ± 0.7	1.7 (38%)
11 –CLIN2+PROG1+INTRO1	99.8 ± 0.5	96.6 ± 4.7	87.2 ± 11.2	12.5 ± 1.7	12.0 (96%)
12 –CLIN2+PROG1	99.8 ± 0.6	95.8 ± 5.7	84.9 ± 12.6	9.1 ± 1.2	8.6 (95%)
13 –CLIN2+PROG2+INTRO1	99.8 ± 0.5	96.3 ± 5.1	85.4 ± 11.2	9.5 ± 1.4	9.0 (95%)
14 –CLIN2+PROG2	99.8 ± 0.6	95.6 ± 5.8	83.5 ± 12.6	6.1 ± 0.9	5.6 (92%)
15 –CLIN2+PROG3+INTRO1	99.2 ± 1.9	92.6 ± 9.6	79.1 ± 15.7	9.2 ± 1.3	8.6 (94%)
16 –CLIN2+PROG3	98.9 ± 2.4	91.1 ± 11.1	76.2 ± 18.1	5.8 ± 0.9	5.3 (91%)
17 –CLIN2+PROG4+INTRO1	100.0 ± 0.0	99.6 ± 1.0	96.0 ± 5.5	9.1 ± 1.4	8.6 (94%)
18 –CLIN2+PROG4	100.0 ± 0.0	98.4 ± 1.5	95.4 ± 6.3	5.8 ± 0.9	5.3 (91%)
19 –CLIN2+PROG5+INTRO1	98.9 ± 2.1	91.7 ± 10.2	77.9 ± 15.9	4.9 ± 0.7	4.4 (89%)
20 –CLIN2+PROG5	98.6 ± 2.8	89.5 ± 12.4	73.0 ± 19.0	2.3 ± 0.4	1.7 (76%)

^a^ P_H_*, design prevalence

^b^ % of total surveillance cost

**Fig 4:**
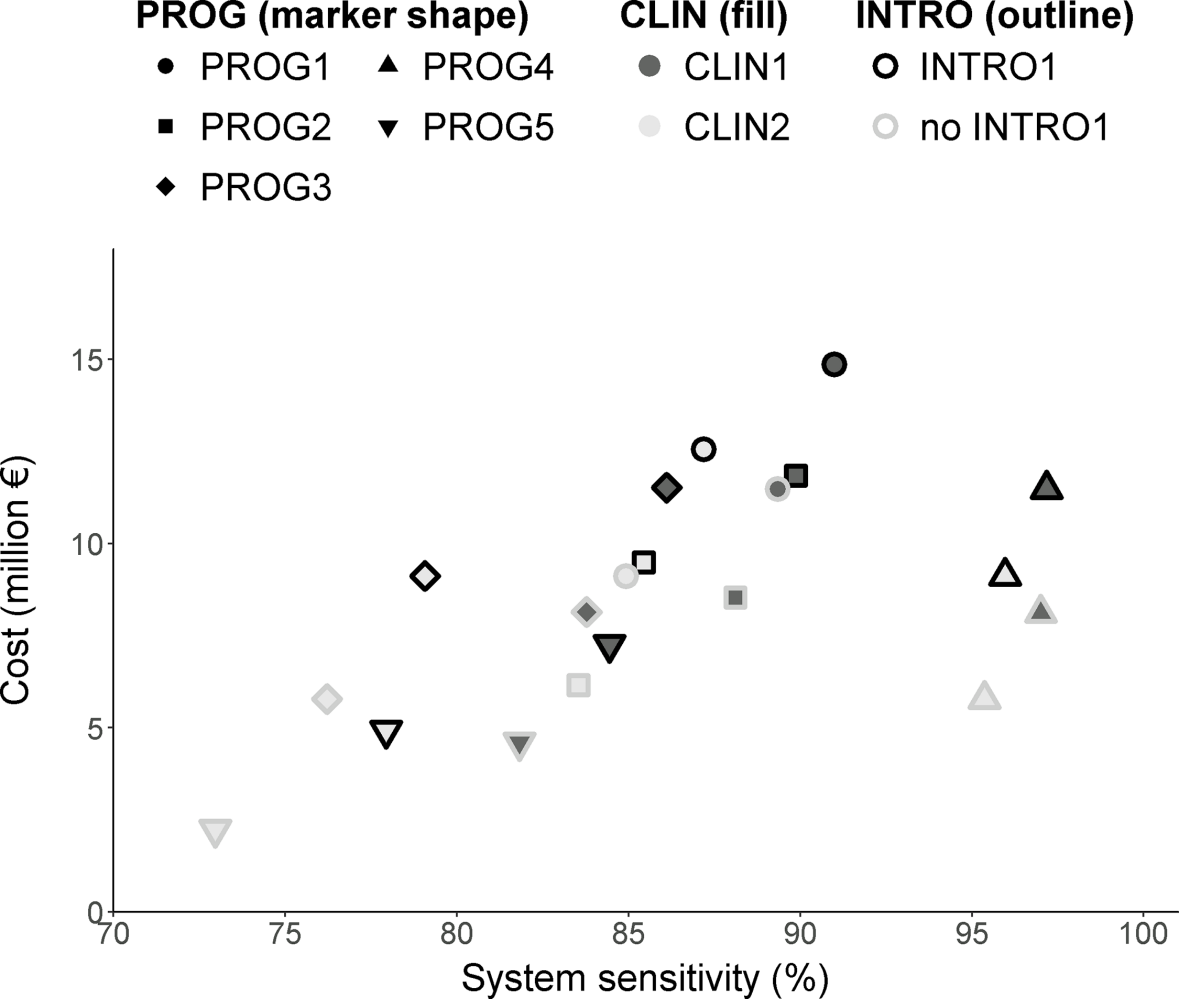
Relationship between surveillance system sensitivity and cost-effectiveness for bovine brucellosis in France at P = 0.01%. Each scenario included programmed serological testing (PROG, described by marker shape), clinical surveillance of abortions (CLIN, described by marker fill), and testing at introduction or not (INTRO, described by marker outline color).

All alternative scenarios were less expensive in terms of surveillance cost than the current system, but not necessarily as effective (Table 4). Five scenarios were predicted to have a mean sensitivity of 90% or above at P_H_* = 0.01%, including scenario 18 (PROG4+CLIN2) with a mean cost of 6 million €, scenarios 8 (PROG4+CLIN1) and 17 (PROG4+CLIN2+INTRO1) with a cost around 8–9 million € and scenarios 7 (PROG4+CLIN1+INTRO1) and 3 (PROG2+CLIN1+INTRO1) with a cost of 12 million € (Fig 4). These costs corresponded to a reduction by 20 to 61% in comparison with the current surveillance system. The cost for farmers, who would pay expenses for PROG surveillance, would be reduced by 25–56%.

For P_H_* = 0.02% or higher, scenario 20 (PROG5+CLIN2+INTRO1) was predicted to be the least expensive for a mean sensitivity of 90% (Fig 4, Table 4), with a reduction in annual surveillance costs by about 85% in comparison with the current system.

### Influence of key parameters’ values on model outputs

Within-herd prevalence was predicted to influence the sensitivity (CSe) of PROG1 (LCC = 0.75, Bonferroni-corrected p-value<0.001) and CLIN1 (LCC = 0.34, Bonferroni-corrected p-value = 0.03). The proportion of farmers notifying abortions influenced the sensitivity of CLIN1 (LCC = 0.75, Bonferroni-corrected p-value<0.001). The sensitivity of INTRO1 was mainly influenced by the annual number of bovine purchases in the high-risk group (LCC = 0.61, Bonferroni-corrected p-value<0.001), the probability of infection in introduced bovine (LCC = 0.41, Bonferroni-corrected p-value = 0.01), the proportion of bovines screened at purchases (LCC = 0.36, Bonferroni-corrected p-value = 0.02) and the proportion of herds subject to control at purchases (LCC = 0.39, Bonferroni-corrected p-value = 0.003). The sensitivity of the bacteriology tests (LCC = 0.42, Bonferroni-corrected p-value = 0.03) influenced the sensitivity of PROG1.

The cost of CLIN1 increased with improving rate of abortion notification (LCC = 0.93, Bonferroni-corrected p-value<0.001). For PROG1, the cost was influenced by the veterinary fee for the herd visit (LCC = 0.59, Bonferroni-corrected p-value<0.001) and the laboratory fee for the BPAT analysis (LCC = 0.59, Bonferroni-corrected p-value<0.001). The cost of INTRO1 was influenced by the annual proportion of introductions in the high-risk herds (LCC = 0.37, Bonferroni-corrected p-value = 0.04) and by the proportion of herds with at least one introduction investigated (LCC = 0.65, Bonferroni-corrected p-value<0.001). The influence of other parameters on model outputs is provided in Table 5.

**Table 5:** Influence of key parameters’ values on the predicted sensitivity (CSe) and cost of each component of the current surveillance system for bovine brucellosis in France.

	CLIN	PROG	INTRO
**CSe**			
Annual number of introductions into the herds of the *high-trade* risk group (N_HT_)	0.04 (1.00)^**a**^	0.02 (1.00)	0.61 (<0.001)
Annual number of introductions into the herds of the *low-trade* risk group (N_LT_)	-0.01 (1.00)	-0.02 (1.00)	0.02 (1.00)
Within-herd prevalence (P_U_*)	0.34 (0.03)	0.75 (<0.001)	-
Probability of infection in introduced bovine	-	-	0.41 (0.01)
Proportion of brucellosis-related abortions (%)	0.10 (1.00)	-	-
Proportion of abortions detected (%)	0.15 (1.00)	-	-
Proportion of notifications/investigations (%)	0.75 (<0.001)	-	-
BPAT sensitivity (%)	0.04 (1.00)	0.16 (1.00)	0.09 (1.00)
CF test sensitivity (%)	0.01 (1.00)	0.26 (0.49)	0.15 (1.00)
Bacteriology sensitivity (%)	0.14 (1.00)	0.42 (0.03)	0.16 (1.00)
Bulk milk ELISA sensitivity (%)	-	0.02 (1.00)	-
Milk ring-test sensitivity (%)	-	0.13 (1.00)	-
Allergic skin-test sensitivity (%)	0.07 (1.00)	0.22 (1.00)	0.07 (1.00)
Annual proportion of introductions investigated per herd (%)	-	-	0.36 (0.02)
Annual proportion of herds with ≥ 1 introduction investigated (%)	-	-	0.39 (0.003)
**Cost**			
Annual number of introductions into the herds of the high-trade risk group (N_HT_)	0.01 (1.00)	-0.04 (1.00)	0.37 (0.04)
Annual number of introductions into the herds of the low-trade risk group (N_LT_)	-0.02 (1.00)	-0.01 (1.00)	0.01 (1.00)
Within-herd prevalence	-0.07 (1.00)	0.02 (1.00)	-
Probability of infection in introduced bovine	-	-	0.02 (1.00)
Proportion of abortions detected (%)	0.23 (0.36)	-	-
Proportion of notifications/investigations (%)	0.93 (<0.001)	-	-
Annual proportion of abortions (all causes included) (%)	0.27 (0.23)	-	-
Laboratory cost (€) for BPAT–abortion investigation	0.04 (1.00)	-	-
Vet fee (€) for visit–other cases	-	0.59 (<0.001)	0.32 (0.12)
Vet fee (€) for commuting time (per km)–other cases	-	0.29 (0.15)	0.13 (1.00)
Vet fee (€) for blood sampling–other cases	-	0.27 (0.29)	0.08 (1.00)
Laboratory cost (€) for BPAT–other cases	-	0.59 (<0.001)	0.15 (1.00)
Laboratory cost (€) for milk ELISA	-	0.14 (1.00)	-
Annual proportion of introductions investigated per herd (%)	-	-	0.24 (0.78)
Annual proportion of herds with ≥ 1 introduction investigated (%)	-	-	0.65 (<0.001)

^a^ Linear correlation coefficient (Bonferroni corrected p-value for multiple tests within each SSC)

## Discussion

To our knowledge, this study presents the first assessment of the efficiency of a bovine brucellosis surveillance system. Other studies, using mathematical modelling of disease spread have evaluated the ability of surveillance systems in detecting (early) any introduction of bovine brucellosis [20, 21], but cost-effectiveness analyses were not included. Our findings make clear that the surveillance system currently in place in France is largely able to meet its objective of detecting at least one case per year at P_H_* = 0.02% or higher, thanks to the high sensitivity of the programmed surveillance combined to the good performance of the surveillance of abortions; in contrast, controls at introduction were predicted to be not effective in detecting disease at such a low design prevalence. Yet, other surveillance systems based on programmed serological screening in a random or risk-based sample of herds and clinical surveillance were predicted to be more efficient (i.e. to provide a similar or higher sensitivity at a lower cost). The assumptions of the models and implications of the findings are discussed with the view to improving the efficiency of the French brucellosis surveillance system.

### Risk of brucellosis introduction

The French cattle population was divided into three risk groups according to the number of introductions (purchases) into the holdings, with a higher risk for herds introducing a large number of bovines [23]. The level of risk was not assumed to depend on the brucellosis status of the country of origin of purchased animals, because the OBF status does not fully guarantee the absence of latent infections; the outbreak in northern France in 2012 resulted from the introduction of an infected bovine from Belgium, which had been OBF since 2003. Other routes of brucellosis introduction were identified, including transmission from wildlife, contacts on pastures at border with non-OBF countries and transmission during insemination, but were not incorporated for the following reasons. First, the spillover of *B*. *melitensis* from a wild Alpine ibex population in France [5] was followed by a large culling of the wild ungulates to decrease population size and brucellosis prevalence. Therefore, the risk of transmission from wildlife has been theoretically strongly reduced and was considered as negligible in the analysis. A tight monitoring of this protected species was advocated for a better knowledge of the population health and contacts with livestock. Seventy-six cattle exploitations are at risk of infection because bovines cohabite with wild ibex in the Alpine pastures, which represents 0.03% of the total number of holdings in metropolitan France [46]. Ongoing field investigations aim to identify other wildlife reservoirs in France and the distribution of wild ungulates sensitive to brucellosis make that only few tens of holdings would be considered as at-risk. Should a risk-based surveillance of bovine brucellosis be implemented in France, including these exploitations into the high risk group would enhance the detection of latent infections, with no marked increase in surveillance expenditures. Second, the risk of brucellosis introduction into French holdings through contacts on pastures with infected cattle from neighboring countries was considered as negligible given that Spain is the only country with non OBF-regions at the border with France and that the contacts on pastures among transhumant herds are strictly regulated. Third, the venereal route is not considered to be epidemiologically important in transmitting brucellosis in cattle and was thus not incorporated into the analysis. Yet, infected semen used in artificial insemination could be an important issue [47]; this is currently strictly controlled in France by regularly testing bulls used for artificial insemination and ensuring that they originate only from OBF herds.

### Surveillance costs

Overall, the current surveillance system (scenario 1) was estimated to cost at least 15 million €, which matches the data-based evaluation of 17 million € for 2013 (based on numbers of vets’ visits, samples and laboratory analyses from the French national animal health information database) [11]. The breakdown was as follow: 10.2 million € for programmed surveillance, 2.7 million € for introduction (purchase) controls, and 3.7 million € for abortion investigations; other marginal expenses (not considered in the scenario tree analysis) included 0.5 million € for surveillance derogations in fattening herds and about 0.03 million € for investigations of suspect cases [11]. Simplifying hypotheses considered in the scenario-tree model made that some costs were somewhat underestimated in comparison to the data-based study. First, only BPAT was considered as a detection test for PROG and INTRO while ELISA on individual serum or on pools of serums are also permitted, with a cost for ELISA on individual serums about 2-to-3 times higher than the BPAT cost [11]. Second, costs for confirmatory analyses and epidemiological surveys in case of positive results were not accounted for, given the low number of positive results.

The costs of administrative management and coordination of the surveillance system and control measures for the Government services or farmers’ animal health organisations, known as *Groupements de Défense Sanitaire* (GDS), which co-ordinate surveillance programs, have never been estimated. Although these costs are not expected to markedly differ among alternative scenarios, the knowledge of human and material resources involved in the functioning of surveillance systems and implementation of control measures is needed to refine the analysis of surveillance efficiency. Besides, capital (or fixed) costs, such as building, maintenance costs or training, have never been evaluated either.

### System efficiency

Our findings indicate that, in comparison with the current system, several alternative scenarios would be more efficient (i.e. at least as effective and less expensive). The range of suitable surveillance scenarios depends on both the choice of the minimum expected sensitivity at the design prevalence decided by animal health managers and the European policy framework regarding bovine brucellosis surveillance and control. We used a P_H_* = 0.01% to compare scenarios, which is 20-times lower than the threshold prevalence set by the European regulations for maintaining the OBF status. However, it corresponds to about 20 infected herds which would imply a control cost of several million €; for example, in 2012, 1.7 million € were spent for the two outbreaks.

Considering a minimum expected sensitivity of 90% at P_H_* = 0.01% (as predicted for the current system), suitable alternative scenarios included clinical surveillance (with the notification of all or series of abortions) and either serological screening of all animals (over two years-old) in 20% of beef herds and milk testing in all dairy herds (PROG2) or risk-based sampling of beef and dairy herds (PROG4). Controls at introduction were predicted to have no added value. Fostering the evolution of the surveillance system in one of these directions would be in agreement with the European regulations and farmers perceptions on brucellosis risk and surveillance. First, both PROG2 and PROG4 fulfill the requirements of the European regulation [6], which requires an annual testing of all reproductive animals in at least 20% of herds for at least five years after obtaining the OBF status. Given that France has been OBF for more than five years, an alleviation could be considered. Yet, our findings shows that a screening of 20% of bovines (over two years-old) in 20% of at-risk herds (PROG5) which would further reduce surveillance costs would not be as effective at P_H_* = 0.01%, but could be considered if a lower level of surveillance was accepted (P_H_* = 0.02%).

Second, our findings showed that the current clinical surveillance was very sensitive in detecting at least one case of bovine brucellosis over one year at P_H_* = 0.05%, but at lower P_H_*, its performance suffered from imperfect detection and notification of abortions. We note that the sensitivity of clinical surveillance is influenced by the within-herd prevalence, which depends on *Brucella* strain and type of cattle production, and may thus vary among infected herds [37]. Therefore, clinical surveillance is more effective at detecting brucellosis in case of acute within-herd transmission. The European directive [6] requires farmers in OBF countries to notify any cases of abortion suspected of being due to brucellosis, which implies implicitly that farmers do not have to report abortions when brucellosis is not considered as a probable cause. The notification of a series of abortions over a period of time is already implemented in other European countries and aims at targeting primarily infectious episodes rather than sporadic events [28, 29, 48, 49]. Accordingly, in simulations with CLIN2, the model ignored unique abortion occurrence, which resulted in a lower mean sensitivity (and large variability) in comparison to CLIN1 (assuming no change in the probability of abortion notification). In France, it has been shown that most farmers did not consider brucellosis in the event of a sporadic abortion or if a non-infectious cause was suspected [9]. In consequence, about two-thirds of farmers–who detected abortion(s)–do not notify any [10]; other reasons for not reporting were related to health and economic factors, practical difficulties, and integration in socio-technical networks [9]. Therefore, revising the definition of a suspect case (from one abortion to a series of two abortions within 30 days) would make the clinical surveillance more acceptable for farmers and veterinarians, who are anticipated to be more prone to report abortions if several cows are concerned [9]. Our study indicated that an increase in the notification rate would make the system even more sensitive, though more expensive. Although this change would potentially increase the time elapsed between the date of detection of the first abortion and the date of notification of the series of abortions, the capacity of the system to detect the disease early is currently mainly influenced by the sensitivity of the surveillance system rather than the notification delay [8].

Last, our model indicated that introduction controls were not predicted to be an important component of surveillance because of the low risk of infection in introduced animals (which originate from OBF herds) and the low proportion of purchased animals that are tested. Actually, given that introduced bovines originate from OBF herds, we assumed a low probability of infection for all these animals, although those from holdings with a particular risk may have a higher probability of being infected. We found an influence of this parameter on the sensitivity of INTRO1, suggesting that our model may underestimate the overall sensitivity of scenarios including INTRO1. Besides, the criteria for selecting animals to be tested do not seem to be sufficiently sensitive. Indeed, in 2012, the infected cow introduced from Belgium (before disease was reported there) was exempted from a control at purchase. The European directive does not require introduction controls in OBF countries [6], and our findings showed that replacing the current surveillance system (scenario 1) by a system with risk-based serological screening and abortion surveillance only (i.e., with no control at introduction) was predicted to strongly reduce annual surveillance cost with no loss in sensitivity (in comparison with the current system).

Although such changes in surveillance are predicted to cut the annual surveillance costs by 20–61% (for scenarios providing a minimum predicted sensitivity of 90% at P_H_* = 0.01%), it must be noted that, in France, the bovine brucellosis surveillance system benefits to the surveillance of other diseases, since the operations related to PROG and INTRO are conducted jointly to the surveillance for other health hazards (such as bovine tuberculosis or Infectious Bovine Rhinotracheitis). Thus, it was estimated that about 30% of the veterinary fees for the current brucellosis surveillance system (visits and blood sampling) are shared with other diseases [11]. Therefore, the savings for cattle farmers may be smaller than the expected 25–56% decrease. Besides, the savings will depend on the mode of testing of the herd and the brucellosis risk. With PROG2, holdings tested by bulk milk serology will still be tested annually–for a cost including only the fee for the laboratory analysis (about 4.7 € annually)–while herds tested by blood serology will be tested on average every five years (for a mean annual cost of about 38.5 € for an average-size beef herd versus 65.2 € currently). With PROG4, only high-risk herds will be tested and will thus support the cost of the national-scale surveillance program. A more balanced option would be to share surveillance costs among all farmers, which may be facilitated by the GDS. Furthermore, a part of annual savings (in comparison with the current system) could be re-injected into the system to improve the participation of field actors in the surveillance by providing support, enhancing risk communication, developing adequate diagnostic tools and implementing a national protocol for differential diagnosis of abortions [48]. These improvements are anticipated in the frame of the French Platform for Animal Health Surveillance [49].

## Conclusions

Scenario-tree model provides an effective tool for evaluating the efficiency of surveillance systems and can be easily updated as new information become available or as the brucellosis situation evolves. Combined clinical surveillance and risk-based screening of herds is a cost-effective approach for demonstrating disease freedom in France. This work could easily be adapted and extended to the brucellosis setting and the characteristics of cattle production in other countries, provided that sufficient financial and epidemiological data are available. In a situation of decreasing resources, the knowledge of surveillance costs is an essential prerequisite to conduct rigorous evaluation of ongoing surveillance activities and help stakeholders make decisions about prioritization of measures and resource allocation.
